# Oculo-facio-cardio-dental (OFCD) syndrome: a case report

**DOI:** 10.1186/s13256-023-04244-x

**Published:** 2024-01-04

**Authors:** Tung Thanh Nguyen, Anh Thai Hoang Truong, Vu Anh Hoang, Duong Van Huynh, Tuan Van Nguyen, Chanh Trung Le, Dung Thi Phuong Dang, Minh Huu Nhat Le

**Affiliations:** 1 Department of Maxillofacial Surgery and Dental, Franco-Vietnamese Hospital, Ho Chi Minh City, Vietnam; 2 Department of Maxillofacial Surgery, University of Medicine and Pharmacy, Ho Chi Minh City, Ho Chi Minh City, Vietnam; 3 Center for Molecular Biomedicine, University of Medicine and Pharmacy, Ho Chi Minh City, Ho Chi Minh City, Vietnam; 4 Department of Maxillofacial Surgery, National Hospital of OdontoStomatology, Ho Chi Minh City, Vietnam; 5 Medical Department, Franco-Vietnamese Hospital, Ho Chi Minh City, Vietnam; 6 International Ph.D. Program in Medicine, College of Medicine, Taipei Medical University, Taipei City, Taiwan; 7 Research Center for Artificial Intelligence in Medicine, Taipei Medical University, Taipei City, Taiwan

**Keywords:** OFCD syndrome, Dental anomalies, *BCOR* gene

## Abstract

**Background:**

Oculo-facio-cardio-dental (OFCD) syndrome is a rare condition that affects the eyes, face, heart, and teeth of patients. One notable dental characteristic of OFCD is radiculomegaly, or root gigantism, which highlights the role of dentists in detecting this syndrome. OFCD is an X-linked dominant syndrome that results from a variant in the BCOR gene. Our study presents the first documented case of OFCD in Vietnam and reports a novel BCOR gene variant observed in this case.

**Case presentation:**

A 19-year-old Vietnamese female patient with an extremely long root with an abscess was clinically examined for the expression of OFCDs. The radiograph and the variant in *BCOR* gene were also evaluated. We identified abnormalities in the teeth, as well as ocular, facial, and cardiac features, with radiculomegaly of the canines being a specific symptom for OFCDs. The patient’s genetic analysis revealed a pathogenic heterozygous deletion at intron 11 of the BCOR gene, representing a novel variant.

**Conclusion:**

Oculo-facio-cardio-dental syndrome (OFCD) is an extremely rare condition characterized by abnormalities in the eyes, face, heart, and teeth, often caused by variants in the BCOR gene. Radiculomegaly, or enlarged dental roots, is a key diagnostic feature of OFCD, and early detection is crucial for preventing future dental complications.

## Introduction

Oculo-facio-cardio-dental (OFCD) is a sporadic syndrome (OFCD, MCOPS2; OMIM #300166) involving abnormalities in patients' eyes, face, heart, and teeth. As it was related to dental abnormalities, this condition may often be discovered by dentists.

In 1980, Hayward [1] was the first person to the relationship between a patient’s abnormally long teeth and congenital cataracts. In 1990, Marashi and Gorlin [2] reported three similar cases and hypothesized that abnormal root size and congenital cataracts could be characteristics of a specific syndrome. In 1993, Wilkie *et al*. [3] demonstrated a case of a mother and her daughter presenting with ocular, facial, cardiac, and dental abnormalities. He believed that it was an X-linked dominant disorder. Gorlin *et al*. [4] in 1996 named it OFCD syndrome. To date, there were no more than 100 OFCDs cases have been reported all over the world. In those reports, the most of patients were female. The study conducted by David Ng has demonstrated that OFCD syndrome is associated with a genetic variant located on the X chromosome [5].

Despite the presence of characteristic symptoms affecting the facial structures and teeth of patients, the rarity of the condition may increase the likelihood of misdiagnosis by physicians as other disorders [6, 7].

As a result, this article aims to summarize symptoms of this extremely rare syndrome and report an OFCD—diagnosed female patient with a discovery of a novel gene variant of *BCOR*. This was the first reported case of this syndrome in Viet Nam.

## Case report

The case presents a 19-year-old Vietnamese female patient who came to the Maxillofacial Surgery Department of the National Hospital of Odonto-Stomatology in Ho Chi Minh City with a periapical infection and a fistula that had developed from her left mandibular canine. The patient's past medical history was obtained by interviewing both the patient and her mother, and all patient-specific information was de-identified. Written informed consent was obtained from the patient’s legal guardian for the publication of photographs and any accompanying information for the purpose of this case report.

### Past medical history

Strabismus was detected at six months old. At the age of four, she was diagnosed with congenital cataracts. She underwent surgery to remove her cloudy lenses and had them replaced with new, artificial lenses (IOLs—Intraocular Lenses). At 14 years old, she was diagnosed with congenital cardiac diseases, including ostium secundum atrial septal defect, pulmonary artery hypertension and 4/4 leaky tricuspid valves. Furthermore, upon examining the patient's mother, we observed the presence of strabismus, even though no evidence of radiculomegaly was found on her panoramic radiograph.

There was no family history of any genetic diseases. The patient has two younger sisters with typical development. The patient's mother reported a fever and was hospitalized for one week at 22 weeks gestation. No abnormalities were detected during her mother's antenatal ultrasounds.

### Extra-oral examination

**Fig. 1:**
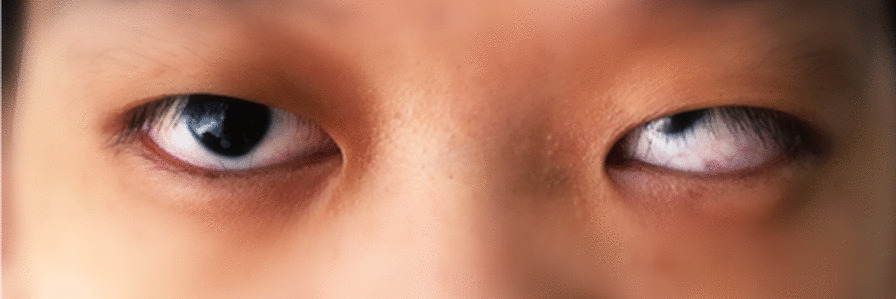
Strabismus, microphthalmia on her left side

**Fig. 2:**
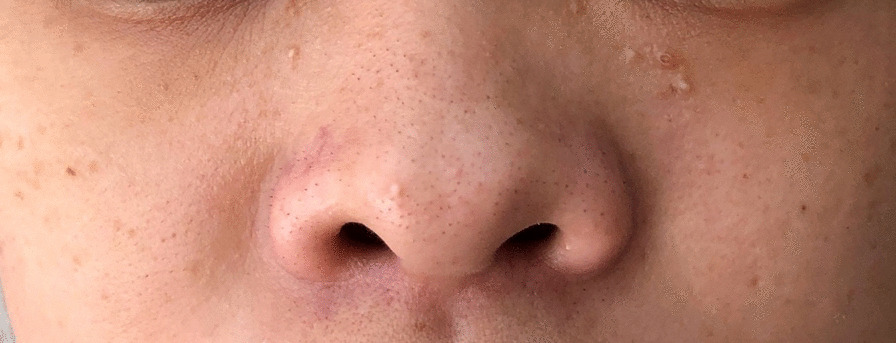
Bifid nasal tip

Clinically, the patient presented with a long and broad face, as well as a concave facial profile in the lateral view. Other physical features included thick eyebrows, a broad and protrusive mandible, and eye abnormalities such as strabismus and microphthalmia on the left side. Visual impairment was present in both eyes, and there were no signs of secondary glaucoma. Additionally, a broad nasal tip with separation of anterior cartilage nasal was observed, along with a fistula on the left chin surrounded by an inflammatory area (Figs. 1, 2). There were no signs of hearing impairment, protruding or hypoplastic ears. The patient had normal pronunciation, but presented with intellectual disability.

### Limb examination

**Fig. 3:**
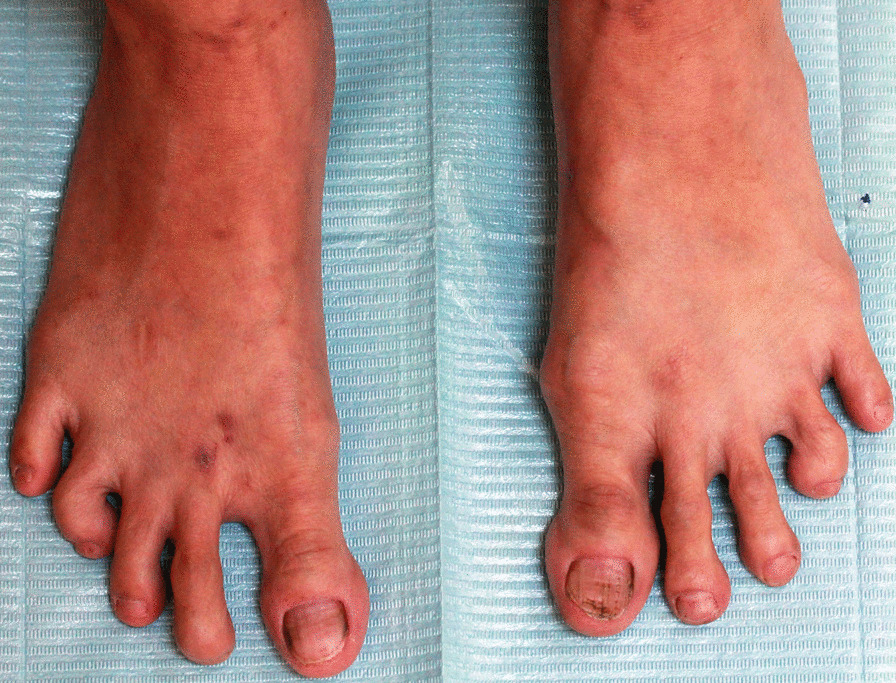
Abnormal foot development (IV—toe camptodactyly and a I—hammer toe)

**Fig. 4:**
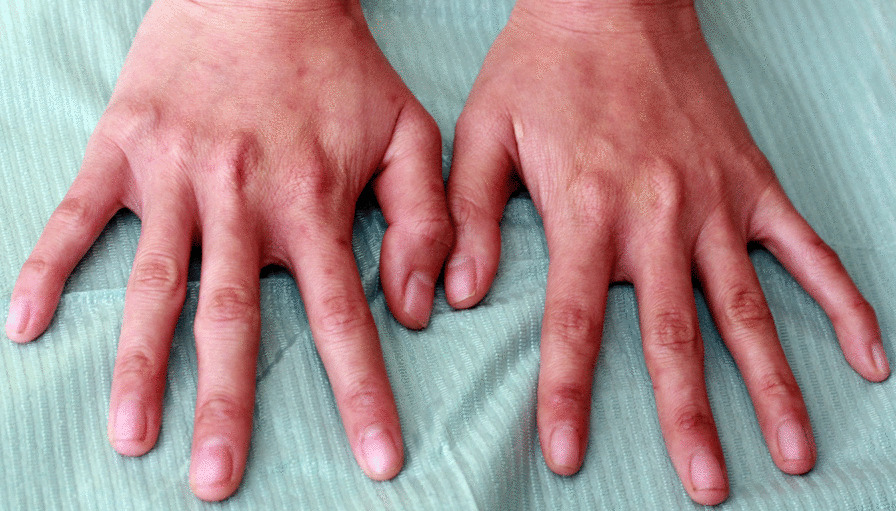
Long and slender fingers

**Fig. 5:**
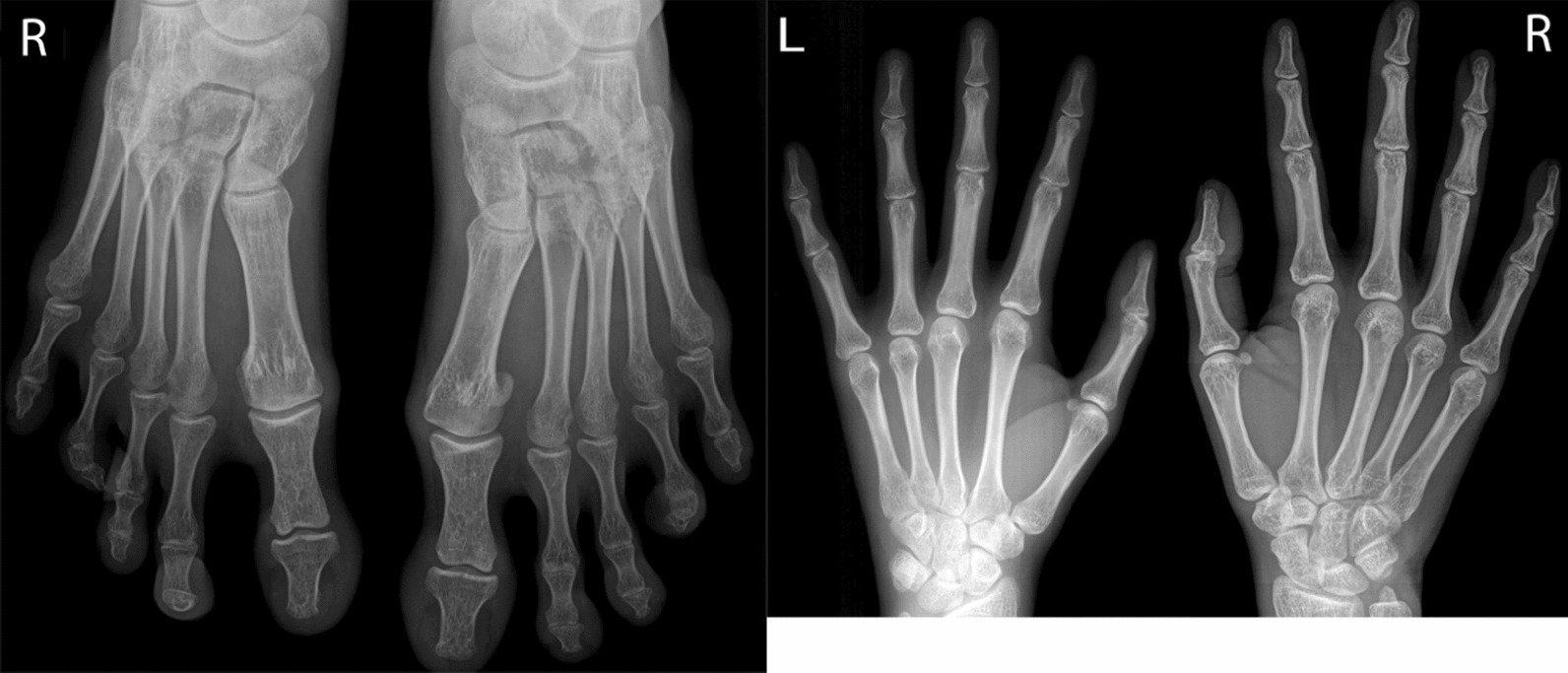
Foot X-ray and hand X-ray

The patient presented with IV-toe camptodactyly and a I-hammer toe. There were no abnormalities detected in her hands (Figs. 3, 4). She did not have hearing impairment, protruding ears, or hypoplastic ears. Her pronunciation was normal, but she had intellectual disability. Radiograph examination revealed IV-toe camptodactyly and I-hammer toe, and her hand radiograph did not show any abnormality (Fig. 5).

### Oral examination

**Fig. 6:**
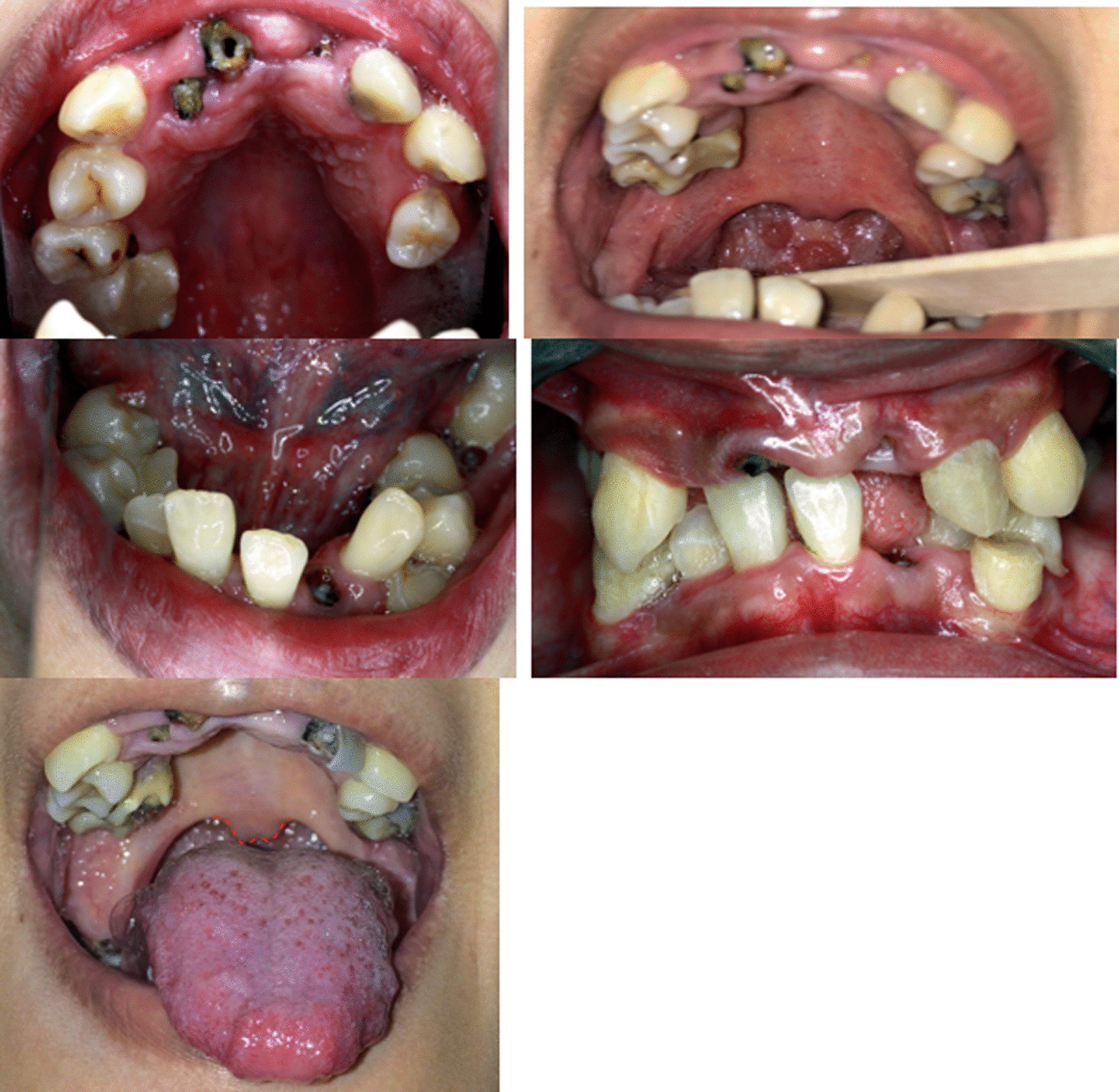
Intra examination showed high arched palate, many dental decays, and mal-occlusion with malposition of teeth 33, 34, 44, 25, heavy dental plaque, stomatitis, glossitis, and bifid uvula was also seen

The patient had a narrow, high-arched palate, with severely crowded teeth and multiple dental caries. Specifically, teeth 11, 12, 16, 21, 26, 32, 33, 36, 43, 46, and 47 had significant decay and required further dental treatments, including extractions and endodontic procedures. Additionally, glossitis was present (Fig. 6).

### Radiograph findings

**Fig. 7:**
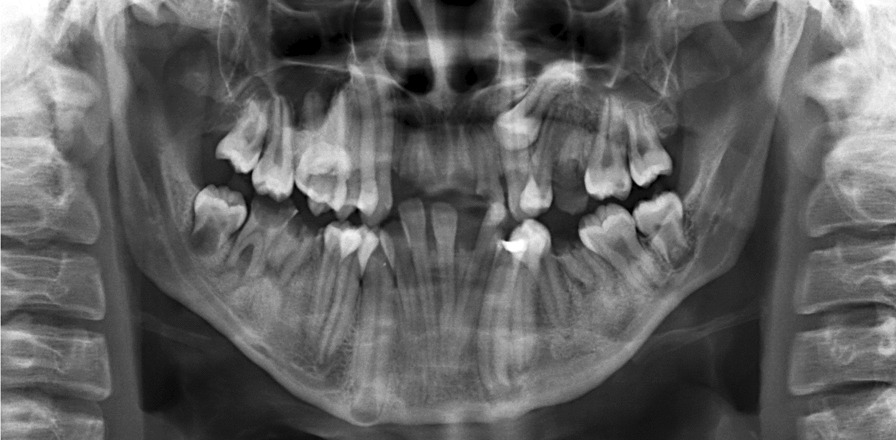
Panoramic panoramic radiograph revealed seriously crowding teeth, many dental caries and radiculomegaly canine with open apices

The Panoramic radiograph revealed a condition of severe crowding of the teeth with malposition of teeth 34, 44, retained roots of teeth 11, 12, 21, 26, 32, 33, 36, 43, 46 with many dental decays. The impacted tooth 24 was also noted. Additionally, the lower premolars and upper right second premolar showed signs of radiculomegaly. Furthermore, the lower anterior teeth had very long roots, and the 37 appeared taurodontic. The 48 was under-developed for 19 years of age. Notably, elongated canines' roots with open apices were demonstrated in all quadrants of her jaws. Moreover, a periapical infection was detected on her left mandibular canine through the panoramic radiograph (Fig. 7).

### Genetic analyses

**Fig. 8:**
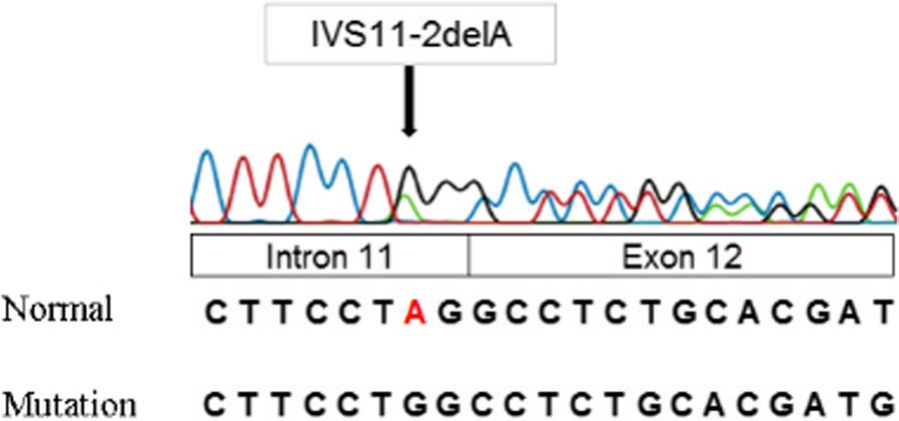
*BCOR* gene sequencing results: detected heterozygous variant IVS11-2delA on intron 11 of *BCOR* gene

Genomic DNA was extracted from peripheral blood using the GeneJET™ Whole Blood Genomic DNA Purification Mini Kit (Thermo Fisher Scientific, MA, USA). Genomic DNA was used to amplify exons 1 through 15 and the exon–intron boundaries of the *BCOR* gene using pairs of PCR primers designed by our own. Purified PCR products were sequenced in both directions using Big Dye Version 3.1 and an ABI 3500 Genetic Analyzer (Applied Biosystems, CA, USA). As a result, the patient was found to be heterozygous for a novel single-base deletion within intron 11 (IVS11-2delA) of the *BCOR* (Fig. 8).

### Diagnosis and treatment

**Fig. 9:**
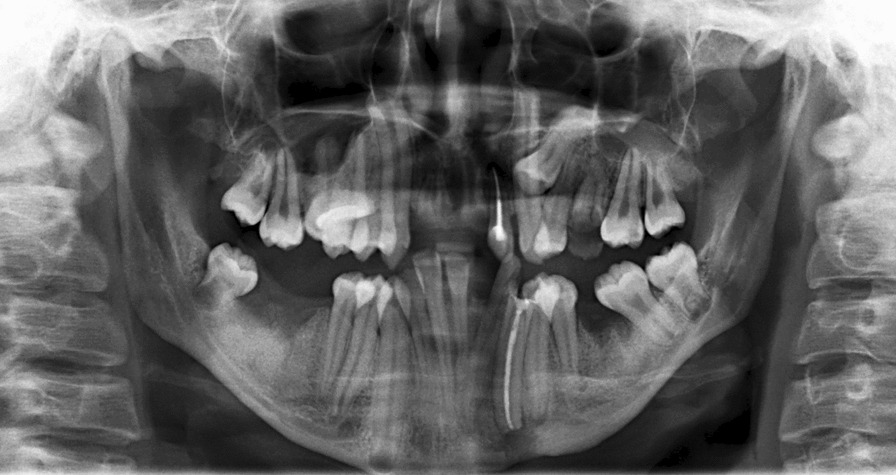
Panoramic film after root canal treatment

**Fig. 10:**
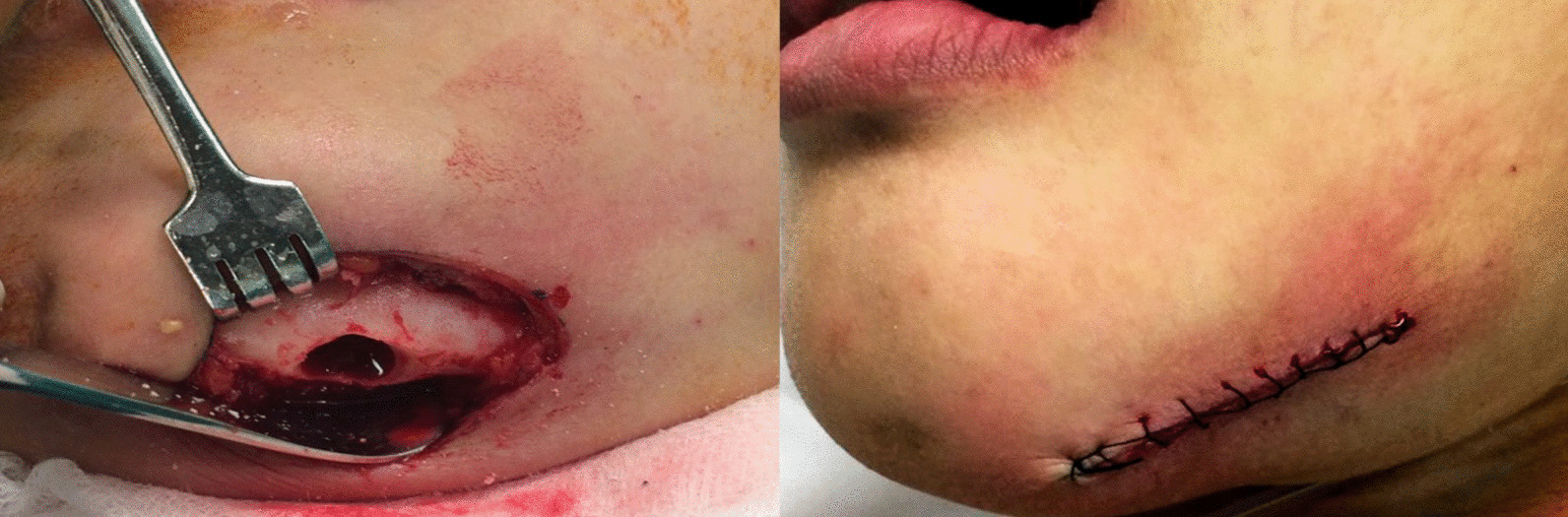
Extraoral apicoectomy with a fistula removal

The patient was diagnosed with periapical infection resulting from her left mandibular canine, and was found to have OFCD syndrome. The treatment plan included a root canal treatment for the affected tooth followed by an extraoral apicoectomy to remove the fistula on her left chin (Figs. 9, 10).

### Follow up

The patient experienced no pain or discomfort in the surgical area. An OPG was taken two weeks post-surgery, and no abnormalities were observed on the X-ray. The patient was re-examined 4 months after surgery and reported no chin pain. At that moment, we did not conduct an X-ray. No adverse events were documented.

## Discussion

OFCD is a rare genetic syndrome with no more than 100 cases reported in literature [8–10], characterized by ocular, facial, cardiac, and dental abnormalities. Early and definitive diagnosis of this syndrome is considered problematic. However, OFCD syndrome has some specific dental symptoms, including an extreme elongation of canine roots and an open apex. Fortunately, these signs could be easily identified on panoramic dental radiograph.

OFCD syndrome has been misdiagnosed as maternal exposure to rubella during pregnancy [6, 11, 12]. However, in some cases, abnormal symptoms in the teeth, particularly radiculomegaly, and the absence of maternal rubella infection during pregnancy, help to differentiate the diagnosis [13]. In addition to the four main symptoms (including ocular, facial, cardiac, and dental), OFCDs patients often have the abnormality in the ears (hearing impairment, protruding ears, hypoplastic ears) [10, 12, 14], extremities (toe syndactyly, hammer toes, radioulnar synostosis) [6, 10, 15] and mental retardation [6, 10, 16]. Numerous cases with several BCOR variants have been reported in the medical literature [8, 9, 13, 14, 17–27] (see Table 1). In our report, the patient's chief complaint was the appearance of a fistula developing from her left mandibular canine. She was then indicated to take a panoramic radiograph, and we found that all her canine teeth had extreme elongation with their apex opening. Clinical examination and medical history demonstrated that this patient had abnormalities in her eyes (congenital cataract, cross-eyed), her face (long and narrow faces, bifid nasal tip) (Fig. 2), and her heart (ostium secundum atrial septal defect, pulmonary artery hypertension, and 4/4 leaky tricuspid valves). We speculated that this patient might have an OFCD syndrome. Therefore, we decided to take a screening for *BCOR* variants. The result showed that a heterozygous deletion variant IVS 11-2delA was detected in intron 11 of gene *BCOR*, a novel variant. With these above symptoms, this patient was planned to receive thorough treatment, including extracting all her root teeth, filling all decayed teeth, undergoing a root canal treatment for the left mandibular canine, and having an extraoral apicoectomy with a fistula removal on her left chin. OFCD is a rare syndrome affecting many organs. Early identification of this disease helps prevent oral complications and endocarditis progression due to caries teeth. As a result, we could combine a wide range of specialties to provide patients with comprehensive care.

**Table 1:** Literature review of case report with BCOR variant

	Current study	Ragge/2018/USA [9]			Kato/2018/Japan [17]	Morgan/2019/USA [18]	Zhang/2019/China [19]	Song/ 2019/Korea [20]	Tsuwaki/2005/Japan [13]	Di-Stefano/2015/Italy [14]
General information	Current case	Case 1	Case 2	Case 3	Case 4	Case 5	Case 6	Case 7	Case 8	Case 9
Age (year)	19	13	21	3	10	10	4	31	16	2
Gender	Female	Female	Male	Female	Female	Female	Female	Female	Female	Female
BCOR variant	IVS11-2delA (c.4596-2delA)	c.2428C > T p.(Arg810*)	c.254C > T p.(Pro85Leu)	c.1209_1210delCC p.(Gln404Alafs*35)	Exon 4 c.265G > A	c.2514del(G)	c.1296delT	n/a	n/a	a de novo heterozygous del Xp11.4
Inheritance	De novo	De novo	Maternal	De novo	Denovo		De novo	De novo		
Ocular										
Microphthalmia	Left side	Bilateral	Bilateral (severe)	Bilateral		Left eye microphthalmia			Bilateral	Right eye
Anophthalmia	No									
Congenital cataract	Bilateral	Bilateral		Bilateral	Bilateral	+	+	+	Bilateral	
Glaucoma	n/a	Unilateral				In the right eye			+	
Strabismus	+									+
Posterior embryotoxon	n/a									
Other	Strabismus, left side				Artificial eye on the left side	Hemangioma near her right eyebrow	Bilateral ptosis, eyebrows curvature; mixed nystagmus; right eye exohypertropia in primary position			Secondary cataract
Craniofacial	Long, broad face, concave facial profile in a lateral view, broad and protrusive mandible, prominent chin					no				
Midface hypoplasia	+		+							
Nasal anomalies	Broad nasal tip with separation of anterior nasal cartilage, bifid nasal tip		+		Broadening of the nasal tip		Broad nasal tip	Nasal tip was prominent and bulbous	Broad nasal tip separated nasal cartilage	Broad nasal tip, depressed nasal bridge
Ear anomalies	n/a		+	+	Preauricular tag		Protruding ears	No	Hearing on the right side is impaired slightly	Depressed nasal bridge
Cleft palate	Bifid uvula				–		+	no	no	
High arched palate	+			+			+			
Other	Thick eyebrows, narrow palate, and mandible, small mouth		Downslanting palpebral fissures, long face, tall forehead, thick eyebrows, Long Philtrum		Elongated, biprotrusive, with a thick lower lip		Flat and slightly long, dovetail-shaped uvula			Broad forehead, bifid uvula
Cardiac										
ASD	Ostium secundum atrial septal defect					+	no	+		+
VSD	n/a						no			+
Other	Pulmonary artery hypertension, 4/4 leaky tricuspid valves		Triple heart sounds							Patent ductus arteriosus (PDA), persistent left superior vena cava
Dental										
Late eruption of first teeth	n/a	+		+	n/a					
Impacted teeth	Tooth 24				Tooth 23 was retracted and extruded					
Delayed loss of primary dentition	n/a	+							+	
Radiculomegaly	Canines, premolars, and lower anterior teeth									
Tautodontism	Tooth 37							Teeth 13, 22, 23, 33, 34, 43, 44		
Fused incisors	No									
XQ										
Other	Crowding with malposition of teeth 34,44, many dental caries (11,12,16,21,26,32,33,36,43,46,47) and periapical infection of tooth 33 with skin leaking	Double row of teeth	Recurrent dental infections	Abnormal crown canines + incisors	Malocclusion, anterior dental crowding, canines, central incisors, and first premolars had Open apex	Long roots of her teeth with one missing tooth and first primary tooth loss at 6–7 years of age	Crossbite	Enamel hypoplasia, crown malformation	Lateral crossbite, Oligodontia, enamel hypoplasia,	
	Glossitis, stomatitis, heavy dental plaque, and calculus									
Skeletal										
Hands	Long and slender finder	Long Finger	5th Finger clinodactyly, long Finger	Long Finger		n/a	Short	No		Clinodactyly of the fifth finger
Feet	IV-toes camptodactyly, I-hammer toes, long toes	Long toes			II-hammer toes	n/a	Hammer-type big toes; flexion deformity (2–4 toes of right foot and 2–3 toes of left foot)	No	I-long and wide toe, hammer-type flexion of toes 2 through 4	Syndactyly 2–3, hammer toe of the second
Other	n/a		Scoliosis				Asymmetry of hand size		Forearm on the right side was slightly shorter	
Developmental										
ID	No intellect defect					n/a				
Motor delay	Mental retardation		+		n/a	n/a	No			No
Speech delay	n/a		+		n/a	n/a				No
MRI findings	n/a				n/a	n/a	n/a			No
Lipomatous lesion	n/a		N		n/a	n/a	n/a			
Other				Moderate BA, broad lateral ventricles						
Other findings										
GU anomalies	n/a	Urethral hypoplasia, renal dysplasia, renal, failure, VUR	Cryptorchidism, vesicoureteric reflux, primary enuresis		n/a	n/a				n/a
Other		Hypotonia	Primary enuresis	Stage III T-cell lymphoma			Bilateral papilloma of choroid plexus (PCP), supratentorial hydrocephalus			

	Current study	Mc Govern/2006/Ireland [21]	Atiq/2012/USA [22]	Danda/2014/India [23]		Martinho/2019/Portugal [8]	Türkkahraman/2006/Turkey [24]	Verm/2014/India [25]	Zhou/2018/USA [26]	Zhu/2015/China [27]
General information	Current case	Case 10	Case 11	Case 12	Case 13	Case 14	Case 15	Case 16	Case 17	Case 18
Age (year)	19	8 days	39	8	6	26	15	24	5 weeks	7 months
Gender	Female	Female	Female	Female	Female	Female	Female	Female	Female	Male
BCOR variant	IVS11-2delA (c.4596-2delA)	n/a		c.3490C > T (p.R1164*					p.R1163X (c.3487 C[T)	p. R540Q
Inheritance	De novo	Maternal		Heterozygous						Missense mutation
Microphthalmia	Left side	+						+	+	
Anophthalmia	No									
Congenital cataract	Bilateral		+	+	+	+	+	+		
Glaucoma	n/a							+	No	+
Strabismus	+									
Posterior embryotoxon	n/a									
Other	Strabismus, left side	Deep-set eyes, short palpebral fissures								
Craniofacial	Long, broad face, concave facial profile in a lateral view, broad and protrusive mandible, prominent chin									
Midface hypoplasia	+							+		
Nasal anomalies	Broad nasal tip with separation of anterior nasal cartilage, bifid nasal tip								Broad nasal tip flat nasal bridge	
Ear anomalies	n/a								Left hearing impairment slightly low-set ears	
Cleft palate	Bifid uvula								+	
High arched palate	+			+	+					
Other	Thick eyebrows, narrow palate, and mandible, small mouth		Extended long canine teeth, and nasal changes	High forehead	High nasal bridge	Class II malocclusion on a Class III skeletal base with a prognathic mandible, increased facial proportions, and facial asymmetry long, narrow face	Long and narrow face, high nasal bridge, broad nasal tip with separated cartilages, and a long philtrum eyebrows were laterally curved and thick Class II malocclusion with an extremely deep overbite	Deeply set eyes and a broad nasal tip long and narrow face Class III malocclusion with a negative overjet and deep overbite		
Cardiac										
ASD	Ostium secundum atrial septal defect	+	-				+		+	
VSD	n/a	+	-	+			+	+		
Other	Pulmonary artery hypertension, 4/4 leaky tricuspid valves	Pulmonary valve stenosis	Mitral valve prolapse	Double outlet right ventricle, pulmonary stenosis		Prolapsed mitral valve			Patent ductus arteriosus (PDA)	
Dental										
Late eruption of first teeth	n/a			+	+		+	+		
Impacted teeth	Tooth 24					Gummy smile, and crowded teeth	Extremely long roots and open apices			
Delayed loss of primary dentition	n/a			+				+		
Radiculomegaly	Canines, premolars, and lower anterior teeth					+				
Tautodontism	Tooth 37									
Fused incisors	No									
XQ				Teethskeletal class I with severe vertical growth pattern, increased gonial angle, steep mandibular plane with retroclined incisors, and competent lips				Permanent teeth with extremely long roots and open apices. The roots of maxillary canines were in relation with the inferior border of the orbits and the lower canine roots almost reached the lower border of the mandible The maxillary left central incisor had dilacerated root; all four third molars were congenitally missing		
Other	Crowding with malposition of teeth 34,44, many dental caries (11,12,16,21,26,32,33,36,43,46,47) and periapical infection of tooth 33 with skin leaking			Bifid uvula		Numerous missing teeth The first upper left molar, upper right canine, upper left lateral incisor, first upper left premolar, first upper right molar, first lower right molar, and first lower left molar are absent				
	Glossitis, stomatitis, heavy dental plaque, and calculus									
Skeletal										
Hands	Long and slender fingers			Camptodactyly of the 4th and 5th fingers (right > left), proximally placed thumbs, restricted supination, and pronation of the left forearm, camptodactyly and syndactyly of 2nd and 3rd toes, and sandal gap Elbow radiographs at infancy showed left radioulnar synostosis		Misalignment		Fingers are normal		
Feet	IV-toes camptodactyly, I-hammer toes, long toes			Sandal gaps, syndactyly and camptodactyly of toes	Sandal gap between the 1st and 2nd toes	Valgus foot		Syndactly of 2nd and 3rd toes	Right clubfoot, and bilateral 2–3 toe syndactyly	
Other	n/a	Misalignment of right second toe		Short stature was observed (122 cm; < 3 SD)	Short stature (111 cm, < 3 SD)					
Developmental		No								
ID	No intellect defect						+		+	
Motor delay	Mental retardation								+	
Speech delay	n/a								+	
MRI findings	n/a									
Lipomatous lesion	n/a									
Other										
Other findings										
GU anomalies	n/a	No								
Other		Anterior positioning of the anus	Several episodes of hypoglycemia several episodes of mental confusion associated with a blood glucose level of less than 40 mg/dL reactive lymph node rare congenital disorder				Tall stature	Umbilical hernia at birth		CHD structural brain anomalies Axenfeld–Rieger syndrome, Lenz microphthalmia syndrome, and oculo-facio-cardio-dental (OFCD) syndrome

*BA* brain atrophy, *ASD* atrial septal defect, *VSD* ventricular septal defect, *ID* Intellectual delay, *Cm* centimeter, *SD* standard deviation, *MRI *magnetic resonance imaging, *VUR* vesicoureteric reflux, *CHD* congenital heart defects, *BCOR variant* The BCOR gene is responsible for coding the BCL6 corepressor protein, *N/a* not applicable or not available, *PDA* patent ductus arteriosus, *N* normal

## Conclusion

Radiculomegaly is a crucial dental symptom that is highly specific to OFCDs. This syndrome is often detected by dentists during dental panoramic radiograph examinations. Therefore, dentists play an essential role in identifying and diagnosing this OFCD syndrome. Dentists should identify patients who may have the condition and refer them to geneticists for further examination and testing. In other words, patients need an accurate diagnosis to receive prophylactic treatment for other related conditions.

## Data Availability

The authors affirm that all data available to sustain the report's interpretations were included in the publication and its supplementary materials.

## References

[1] Hayward J (1980). Cuspid gigantism. Oral Surg Oral Med Oral Pathol.

[2] Marashi AH, Gorlin RJ (1990). Radiculomegaly of canines and congenital cataracts—a syndrome?. Oral Surg Oral Med Oral Pathol.

[3] Wilkie A, Taylor D, Scambler P, Baraitser M (1993). Congenital cataract, microphthalmia and septal heart defect in two generations: a new syndrome?. Clin Dysmorphol.

[4] Gorlin RJ, Marashi AH, Obwegeser HL (1996). Oculo-facio-cardio-dental (OFCD) syndrome. Am J Med Genet.

[5] Ng D, Thakker N, Corcoran CM, Donnai D, Perveen R, Schneider A, Hadley DW, Tifft C, Zhang L, Wilkie AO (2004). Oculofaciocardiodental and Lenz microphthalmia syndromes result from distinct classes of mutations in BCOR. Nat Genet.

[6] Gorlin RJ, Cohen MM, Hennekam RC (2001). Syndromes of the head and neck.

[7] Hu Q, Mai J, Xiang Q, Zhou B, Liu S, Wang J (2022). A novel deletion mutation in the BCOR gene is associated with oculo-facio-cardio-dental syndrome: a case report. BMC Pediatr.

[8] Martinho J, Ferreira H, Paulo S, Paula A, Marto C-M, Carrilho E, Marques-Ferreira M (2019). Oculo-facio-cardio-dental syndrome: a case report about a rare pathological condition. Int J Environ Res Public Health.

[9] Ragge N, Isidor B, Bitoun P, Odent S, Giurgea I, Cogné B, Deb W, Vincent M, Le Gall J, Morton J (2019). Expanding the phenotype of the X-linked BCOR microphthalmia syndromes. Hum Genet.

[10] Smith MH, Cohen DM, Bhattacharyya I, Islam NM, Kashtwari D (2018). Radiculomegaly: a case report of this rare dental finding with review of the associated oculo-facio-cardio-dental syndrome. Oral Surg Oral Med Oral Pathol Oral Radiol.

[11] Barthelemy I, Samuels L, Kahn DM, Schendel SA (2001). Oculo-facio-cardio-dental syndrome: two new cases. J Oral Maxillofac Surg.

[12] Kawamoto T, Motohashi N, Ohyama K (2004). A case of oculo-facio-cardio-dental syndrome with integrated orthodontic-prosthodontic treatment. Cleft Palate Craniofac J.

[13] Tsukawaki H, Tsuji M, Kawamoto T, Ohyama K (2005). Three cases of oculo-facio-cardio-dental (OFCD) syndrome. Cleft Palate Craniofac J.

[14] Di Stefano C, Lombardo B, Fabbricatore C, Munno C, Caliendo I, Gallo F, Pastore L (2015). Oculo-facio-cardio-dental (OFCD) syndrome: the first Italian case of BCOR and co-occurring OTC gene deletion. Gene.

[15] Opitz C, Horn D, Lehmann R, Dimitrova T, Fasmers-Henke K (1998). Oculo-facio-cardio-dental (OFCD) syndrome. J Orofac Orthop.

[16] Hilton E, Johnston J, Whalen S, Okamoto N, Hatsukawa Y, Nishio J, Kohara H, Hirano Y, Mizuno S, Torii C (2009). BCOR analysis in patients with OFCD and Lenz microphthalmia syndromes, mental retardation with ocular anomalies, and cardiac laterality defects. Eur J Hum Genet.

[17] Kato J, Kushima K, Kushima F (2018). New radiological findings and radiculomegaly in oculofaciocardiodental syndrome with a novel BCOR mutation: a case report. Medicine.

[18] Morgan T, Colazo J, Duncan L, Hamid R, Joos K (2019). Two cases of oculofaciocardiodental (OFCD) syndrome due to X-linked BCOR mutations presenting with infantile hemangiomas: phenotypic overlap with PHACE syndrome. Case Rep Genet.

[19] Zhang J, Jia H, Wang J, Xiong Y, Li J, Li X, Zhao J, Zhang X, You Q, Zhu G (2019). A novel deletion mutation, c. 1296delT in the BCOR gene, is associated with oculo-facio-cardio-dental syndrome. Sci China Life Sci.

[20] Oh SH, Kang JH, Kang JH, Seo Y-K, Lee SR, Choi Y-S, Hwang E-H (2019). Radiculomegaly of canines in oculofaciocardiodental syndrome. Oral Radiol.

[21] McGovern E, Al-Mudaffer M, McMahon C, Brosnahan D, Fleming P, Reardon W (2006). Oculo-facio-cardio-dental syndrome in a mother and daughter. Int J Oral Maxillofac Surg.

[22] Atiq M, Gong Y, Raju GS, Lee JH (2012). Pancreatic endocrine microadenomatosis in a patient with oculofaciocardiodental (OFCD) syndrome. Pancreas.

[23] Danda S, Van Rahden VA, John D, Paul P, Raju R, Koshy S, Kutsche K (2014). Evidence of germline mosaicism for a novel BCOR mutation in two Indian sisters with oculo-facio-cardio-dental syndrome. Mol Syndromol.

[24] Türkkahraman H, Sarıoğlu M (2006). Oculo-facio-cardio-dental syndrome: report of a rare case. Angle Orthod.

[25] Verma G, Singh GK, Tandon P, Verma SL (2014). A rare syndrome with unusual dental findings: oculo-facio-cardio-dental syndrome. J Oral Maxillofac Pathol JOMFP.

[26] Zhou Y, Wojcik A, Sanders VR, Rahmani B, Kurup SP (2018). Ocular findings in a patient with oculofaciocardiodental (OFCD) syndrome and a novel BCOR pathogenic variant. Int Ophthalmol.

[27] Zhu X, Dai F-R, Wang J, Zhang Y, Tan Z-P, Zhang Y (2015). Novel BCOR mutation in a boy with Lenz microphthalmia/oculo-facio-cardio-dental (OFCD) syndrome. Gene.

